# Strategies for Reducing the Impact of Cycling on the Perineum in Healthy Males: Systematic Review and Meta-analysis

**DOI:** 10.1007/s40279-020-01363-z

**Published:** 2020-10-19

**Authors:** Kamil Litwinowicz, Marcin Choroszy, Anna Wróbel

**Affiliations:** 1 Department of Medical Biochemistry, Wroclaw Medical University, ul. Chalubińskiego 10, 50-368 Wroclaw, Poland; 2 Department of Microbiology, Wroclaw Medical University, ul. Chalubińskiego 4, 50-368 Wroclaw, Poland; 3 Department of Psychiatry, Wroclaw Medical University, Wybrzeze L. Pasteura 10, 50-367 Wroclaw, Poland

## Abstract

**Introduction:**

Perineal pressure associated with bicycle riding is the cause of several genitourinary pathologies, most notably Alcock’s syndrome and subsequent perineal numbness. The possible link between cycling-induced perineal numbness and erectile dysfunction makes the development of strategies for perineal protection in bicycle users critical.

**Objective:**

To assess the effectiveness of strategies for reducing the impact of cycling on the perineum in healthy males.

**Methods:**

We have conducted a systematic review and a meta-analysis of studies examining various means of reducing the impact of cycling on the perineum under the PRISMA guidelines.

**Results:**

Out of 2217 screened studies, 22 met our inclusion criteria, and 6 qualified for meta-analysis. The strategies included various designs of saddles, changes in the cycling position, seat shock absorber, shorts with different padding, using the recumbent bike. Using the no-nose saddle and recumbent bike resulted in a significant reduction of perineal pressure and higher penile oxygen pressure compared with a standard saddle. Indirect evidence supports the protective effect of standing on the pedals every few minutes during cycling. More evidence is needed to support—or dismiss—other strategies.

**Conclusions:**

Current evidence supports the use of no-nose saddles as a mean to reduce the negative impact of cycling on the perineum in healthy males at the cost of worse stability and increase of posterior seat pressure. Standing on the pedals every ten minutes might be an effective and potentially widely applicable strategy. The use of a recumbent bike appears to protect the perineum, but several concerns prevent its widespread use.

**Electronic supplementary material:**

The online version of this article (10.1007/s40279-020-01363-z) contains supplementary material, which is available to authorized users.

## Key Points

**Table:** 

Currently, there is limited evidence regarding the safety of using a no-nose saddle and recumbent bike for perineal protection in healthy male cyclists.
More research is needed to develop optimal guidelines regarding standing on the pedals as a strategy for reducing the impact of cycling on the perineum.
Using the no-nose saddle, standing on the pedals every few minutes and using the recumbent bike are effective in protecting the perineum while cycling.

## Introduction

Cycling is one of the most commonly used cardiovascular exercises. Its health benefits range from reduction of all-cause mortality [1] to improvement of cognitive function [2]. While cycling has significant benefits to health and fitness, the constant pressure exerted by the bicycle seat might be the cause of several complaints, ranging from saddle sores to more serious complaints related to the urogenital system. Schrader et al. [3] examined the effect of riding a bicycle on nocturnal penile tumescence. They have reported a significant, inverse correlation between the pressure exerted on the nose of the bicycle seat and the percentage of sleeping time with an erection. A survey of 2774 cyclists and 1158 non-cyclists revealed that cycling is associated with a significantly higher risk of experiencing perineal numbness and developing urethral stricture [4]. Up to 91% of bicycle users experience perineal numbness [5]. Cyclists complaining of perineal numbness are more likely to report erectile dysfunction (ED) [6, 7]. The link between cycling and ED is still a matter of discussion. A recent meta-analysis by Gao et al. found a positive correlation between ED and cycling when controlling for age (odds ratio [OR] 1.55). However, included studies presented significant heterogeneity [8].

Commonly genital numbness is attributed to Alcock’s syndrome, a condition first described in two cyclists, who suffered from genital hypesthesia lasting over 4 weeks [9]. Vascular occlusion and subsequent hypoxemia of the pudendal nerve may also play a role. Both of these mechanisms can stem from an increase of perineal pressure caused by sitting on the bicycle seat [10]. Nanka et al. proposed that the most important site of compression could be the sulcus nervi dorsalis penis, which courses near the pubic symphysis [11]. This hypothesis is supported by three-dimensional models of the perineum, which point to the area in proximity of pubic symphysis as the most susceptible to the increase in seat pressure [12–14]. This leads to the conclusion that reduction of anterior seat pressure may be the most important factor in reducing the incidence of perineal numbness.

Currently, very little evidence-based advice can be offered for patients complaining of cycling-induced perineal numbness and other conditions associated with high seat pressure. The possible link between perineal numbness and ED highlights the importance of perineal protection. With this in mind, the goal we have set for this systematic review is to present currently available options for mitigating the negative effects of cycling on the perineum, examining how strong the evidence supporting a given strategy is and assessing the size of its effects in healthy males. Additionally, we have reviewed how various strategies impact the comfort of the user and other regions in contact with the seat.

## Methods

The study adhered to guidelines outlined in the Preferred Reporting Items of Systematic reviews and Meta-Analyses (PRISMA) [15].

### Search Strategy

A comprehensive literature search was performed. Our search terms were was (cycling or bicycling or bicycle or cyclist* or bicyclist*) AND ("erectile dysfunction" OR "sexual dysfunction" OR impotence OR perineum OR discomfort OR "seat pressure" OR "saddle pressure" OR “perineal pressure” OR "urethral stricture" OR "saddle sore" OR "genital numbness" OR "genital pain" OR “perineal numbness” OR “perineal pain”) for MEDLINE on PubMed. To account for differences in the search syntax, we have appropriately modified the search terms for other databases. The search terms were applied to the following databases: MEDLINE (1948 to August 2020), Scopus (1970 to August 2020), PEDro (1929 to August 2020), CINAHL (1982 to August 2020) and the Cochrane Central Register of Controlled Trials (CENTRAL, August 2020). Additionally, we have performed a manual search of the references of retrieved articles. The last search was run on August 12th, 2020. Before proceeding with the selection of eligible studies, all duplicates were removed.

### Inclusion and Exclusion Criteria

We have included studies examining the effect of different saddle types, other equipment, and positions while cycling on pressures in various seat regions (including perineal pressure), validated diagnostic questionnaires related to sexual disorders, penile hemodynamics, and subjective complaints related to the perineum. Only human studies were included, and no language restrictions were applied. No publication-status restrictions were imposed. Only studies examining healthy males were included. Studies in which both males and females were examined were included only if the male subgroup had been extracted. The comparator groups consisted of healthy adult males using a conventional cycling position and equipment. We have included only interventional studies. Exclusion criteria included diagnoses of cardiovascular diseases.

### Assessment of Eligibility and Data Extraction

**Fig. 1:**
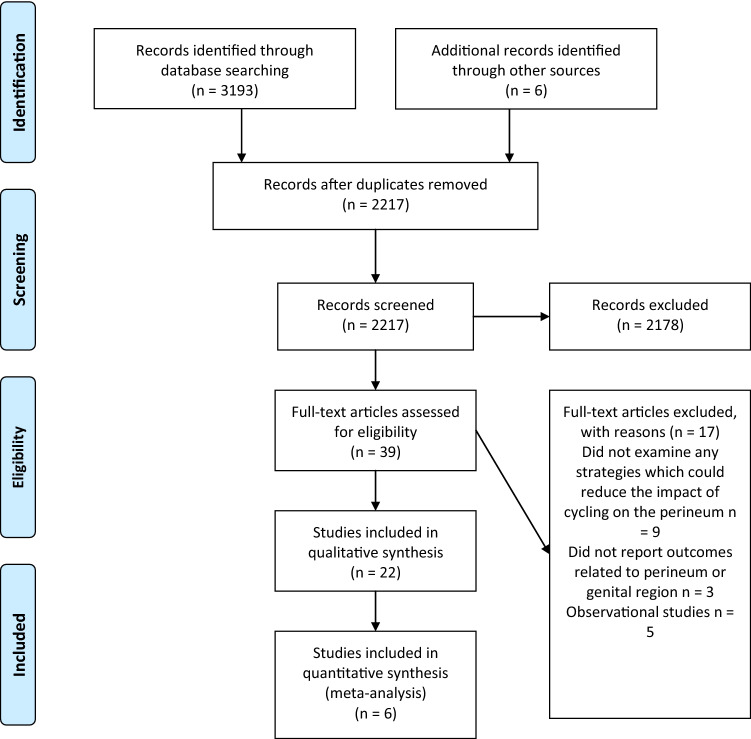
Flow diagram of the study selection process (adapted from PRISMA)

After removing the duplicates, two authors (KL and MC) independently screened obtained studies by titles and abstracts for relevance to the topic of our systematic review. Studies obtained by screening were read in full-text and eligibility based on inclusion and exclusion criteria was determined. Discrepancies were discussed and if disagreement was not resolved, a third author (AW) arbitrated. Only studies published in peer-reviewed journals were included. The eligibility assessment of studies is summarized in Fig. 1.

For data extraction a datasheet with fields relating to study characteristics (title, author, study design, publication year, funding, conflict of interest, comparators), participants (inclusion and exclusion criteria, demographic data), and outcomes was created.

### Risk of Bias

The risk of bias was assessed independently by two reviewers (KL and MC) with ROBINS-I [16] for before-and-after studies and with RoB-2 [17] for cross-over and randomized trials (Electronic Supplementary Material Figs S1 and S2). In case of disagreement, the third author (AW) mediated. Due to the nature of included interventions (e.g. changes of position and different types of saddle), we did not consider the blinding of the participants. Risk of bias plots were generated using the robvis tool [18]. Due to a small number of studies included in meta-analyses, publication bias was not assessed.

### Synthesis of Results

We performed separate meta-analyses for each strategy where we could obtain at least three clinically homogenous studies. We expressed the results as a standardized mean difference with corresponding 95% confidence intervals (CIs). We have used RevMan 5.3 [19] for statistical analysis. The results of meta-analyses were visualized with forest plots. To account for heterogeneity across studies, we have used a random-effects model. We have reported pooled effect sizes for each comparison. In the case of Munarriz et al. [20] we have combined two, randomly allocated subgroups, both comparing right and left cavernosal artery peak systolic velocity (CAPSV) between a standard seat and no-nose seat into one group, as suggested by the Cochrane handbook [21].

## Results

**Fig. 2:**
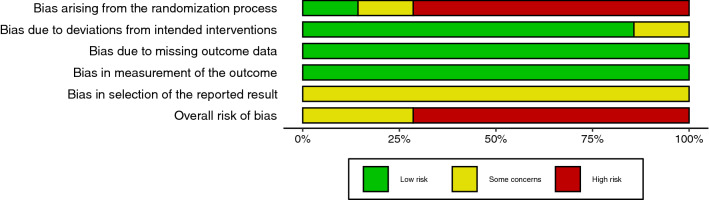
Risk of bias summary plot for cross-over and randomized trials

**Fig. 3:**
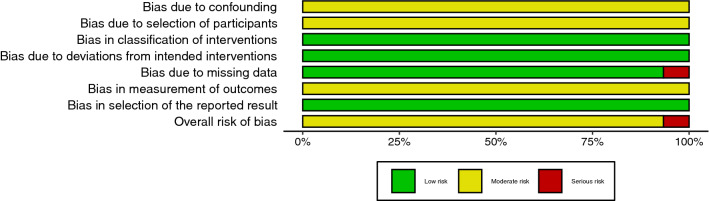
Risk of bias summary plot for before and after studies

The search yielded a total of 3193 results. Chain searching references yielded six additional results. After the removal of duplicates, 2217 studies were assessed for relevance. 39 studies were read in full text and assessed for eligibility using the defined inclusion and exclusion criteria. Out of 39 studies 22 qualified for our systematic review (see Fig. 1 for details) and 6 were used in the meta-analysis (see Table 1 for characteristics of included studies). The risk of bias assessment is summarized in Figs. 2 and 3 (see Electronic Supplementary Material Figs S1 and S2). In total, the studies encompassed 601 participants.

**Table 1:** Characteristics and outcomes of the included studies; N: number of participants

Study	Design	N	Intervention	Outcome measure	Outcomes
Breda et al. [34]	Randomized controlled trial	29	SMP saddle vs standard saddle	Penile oxygen pressure [mmHg]	SMP vs ordinary saddle: Static after 3 min—49.3 vs 25.3 Pedaling after 15 min—52.1 vs 28.5
Bressel and Cronin [36]	Before and after	9	Top handlebar vs drop handlebar position	Mean total, anterior, posterior, left and right seat pressure (kPa)	Top vs drop handlebar: Total seat pressure: 19.3 ± 2.2 vs 17.2 ± 1.4 Anterior seat pressure: 16.3 ± 5.6 vs 15.7 ± 1.8 (non-significant) Posterior seat pressure: 21.1 ± 3.9 vs 17.9 ± 3.2 Left seat pressure: 16.0 ± 2.5 vs 14.4 ± 1.7 Right seat pressure: 16.2 ± 2.2 vs 15.6 ± 1.8
Bressel et al. [25]	Cross-over	17	Standard saddle vs partial cutout saddle vs no-nose saddle	Mean total, anterior and posterior saddle pressure (kPa) Perceived stability	Standard vs partial cutout vs no-nose saddle: Total seat pressure: 27.6 ± 13.48 vs 30.3 ± 14.97 vs 25.3 ± 12.25 (non-significant) Anterior seat pressure: 31 ± 16.08 vs 26.8 ± 15.5 vs 8.99 ± 1.63 Posterior seat pressure: 23.4 ± 15.17 vs 27.1 ± 15.92 vs 27.3 ± 14,68 Perceived stability: 10.2 ± 1.24 vs 9.65 ± 1.19 vs 4.84 ± 1.28
Carpes et al. [41]	Before and after	11	Standard saddle vs holed saddle	Seat pressure (Pa/kg)	Standard vs holed saddle: 2.04 ± 0.5 vs 2.09 ± 0.5
Carpes et al. [24]	Before and after	11	Upright and forward trunk position using a saddle with and without hole	Seat pressure [kPa]	Plain saddle: 69.04 ± 17.9 kPa for trunk at 90° vs 63.38 ± 21.70 for 60° (non-significant) Holed saddle: 66.3 ± 12.92 for 90 and 55.75 ± 23.64 for 60°
Chen and Liu [29]	Before and after	16	Saddles with different protruding node lengths Tops vs drops position	Subjective discomfort levels in the perineum and ischial tuberosity (continuous visual analog scale 10 cm) Subjective stability rated on the continuous visual analog scale (10 cm)	Tops vs drops handlebars: no significant difference in discomfort levels in the perineum and ischial tuberosity Different protruding node lengths: A positive, significant correlation (*r* = 0.994) between protruding node length and discomfort in the perineum; mean VAS score was 2.88 ± 2.11 for a seat without protruding node and 5.02 ± 1.74 for a seat with 12 cm protruding node length A negative, significant correlation (*r* = − 0.914) between protruding node length and discomfort in the ischial tuberosity region; mean VAS score was 5.13 ± 20.3 for a seat without protruding node and 3.85 ± 1.72 for a seat with 12 cm protruding node Short protruding node lengths (0 and 3 cm) were less stable than long protruding node lengths (6–12 cm); Mean stability score was 4.36 ± 2.1 for a seat without protruding node and 2.61 ± 2.08 for a seat with 12 cm protruding node length (lower values signify higher stability)
Cohen and Gross [26]	Before and after	31	Standing vs sitting: 1. Narrow saddle with minimal padding 2. Saddle with a hole in the center 3. Saddle with central depression extending from the back to the front of the seat	Transcutaneous penile oxygen pressure (mmHg)	Different seat designs (non-significant): 9.1 ± 13.8 (narrow with minimal padding) vs 10.2 ± 13.6 (hole) vs 14.7 ± 18.7 (central depression) Standing vs sitting: 38 ± 16.9 vs 11.4 ± 15.5
Jeong et al. [22]	Cross-over	20	1. Narrow unpadded saddle vs wide unpadded saddle 2. Standing vs sitting	Penile blood flow (ml/min/100 g tissue)	84% decrease for narrow saddle and 19% decrease for the wide saddle Standing vs sitting: a reduction from 1.7 to 1.0
Kerstein et al. [23]	Before and after	20	Standing vs sitting	Penile blood pressure obtained using ultrasound Doppler instrument (mmHg)	Standing vs sitting: 126 ± 8 vs 76 ± 9
Lowe et al. [27]	Randomized controlled trial	16	Standard saddle vs no-nose saddle	Perineal and total seat pressure (kPa)	Standard vs no-nose saddle: Mean perineal pressure: 37.2 ± 3.87 vs 19 ± 8.29 Mean total seat pressure: 19.64 ± 2 vs 16.36 ± 3.82
Marcolin et al. [39]	Before and after	9	Shorts with three different pads: basic, intermediate, endurance	Mean total seat pressure and peak perineal pressure (kPa)	BAS vs INT vs END: Mean total seat pressure: 12.6 ± 1.9 vs 12.0 ± 1.7 vs 12.3 ± 1.9 (non-significant) Peak perineal pressure: 46.5 ± 15.1 vs 47.9 ± 15.9 vs 47.9 ± 17.7 (non-significant)
Munarriz et al. [20]	Before and after	33	Standard seat vs seat with no nose	Right/left cavernosal artery peak systolic velocity (CAPSV, cm/s)	Standard vs no-nose seat: 0.36 ± 2.08/0.77 ± 4.45 vs 21.57 ± 11.26/21.13 ± 9.98
Nayal et al. [45]	Before and after	25	Cycling in standing vs sitting position	Transcutaneous penile oxygen pressure (mmHg)	Standing vs sitting: 68 ± 7.6 vs 18.4 ± 4.2
Parthiban et al. [31]	Before and after	20	Standard seat vs seat without nose Road setting vs stationary setting Standard seat vs seat with grooved center channel	Perineal artery occlusion time	Standard vs no-nose saddle: 0.23 occlusion time proportion increase Road setting vs stationary setting: + 0.13 OTP (non-significant) − 0.06 and + 0.06 (two different seats), non-significant
Potter et al. [37]	Before and after	11	Tops vs drops position	Peak anterior and posterior seat pressure	Tops vs drops: Posterior saddle pressure: 0.576 ± 0.596 vs 0.392 ± 0.332 Anterior saddle pressure (non-significant): 0.616 ± 0.504 vs 0.644 ± 0.368
Sanford et al. [38]	Before and after	29	Seatpost shock absorber	Raw pressure changes in anterior and posterior perineum	Stationary with oscillations without shock absorber vs shock absorber (reduction): Anterior perineum: 295 Posterior perineum: 206 Pedaling with oscillation without shock absorber vs shock absorber (reduction): Anterior perineum: 23 (non-significant) Posterior perineum: 29 (non-significant)
Schrader et al. [28]	Before and after	73	Standard seat vs seat without nose after 6 months	Mean perineal pressure (kPa), urogenital numbness, erectile function assessed with Index of Erectile Function Questionnaire	Perineal pressure 20.4 ± 8.9 vs 7 ± 3.2 Urogenital numbness: 73% vs 18% IIEF: 29.12 ± 2.4 vs 29.61 ± 1.35
Schwarzer et al. [30]	Cross-over	20	Standing vs sitting on: 1. Narrow, heavily padded seat 2. Narrow seat with medium padding and V-shaped groove in the saddle nose 3. Wide unpadded leather seat 4. Women’s special wide seat with medium padding and no saddle nose	Transcutaneous penile oxygen pressure (mmHg)	Standing vs sitting: Narrow heavily padded seat: 67.1 ± 13.8 vs 11.8 ± 16.4 Narrow seat with medium padding and V-shaped groove: 75.4 ± 17.1 vs 20.8 ± 19.5 Wide unpadded leather seat: 68.9 ± 18.1 vs 25.3 ± 21.6 (*P* < 0.001) Women’s special wide seat with medium padding and no saddle nose: 78.3 ± 18.4 vs 62.3 ± 20.1 No direct comparison between seats was performed
Sommer et al. [32]	Before and after	100	Standing vs sitting Recumbent bike Narrow saddle vs wide saddle	Transcutaneous penile oxygen pressure (mmHg)	Standing vs sitting: 61.1 ± 7.1 vs 16.8 ± 4.1 Recumbent bike standing vs sitting: 61.1 ± 7.1 vs 59.8 ± 4.2 Narrow vs no-nose saddle: 70% reduction vs 22% reduction in blood flow
Sommer et al. [35]	Cross-over	46	Standing vs sitting in: 1. Reclining position 2. Upright position	Transcutaneous penile oxygen pressure (mmHg)	Standing vs sitting: Reclining position: 61.1 ± 6.9 vs 59.4 ± 3.7 (non-significant) Upright position 60.5 ± 8.1 vs 18.3 ± 5.2
Sommer et al. [44]	Cross-over	40	Standing vs sitting	Transcutaneous penile oxygen pressure (mmHg)	Standing vs sitting: 60.4 ± 7.8 vs 18.4 ± 4
Taylor et al. [33]	Cross-over	15	Standard seat vs experimental seat with a nose cutout	Subjective perineal numbness Objective perineal numbness (Weinstein Enhanced Sensory Testing esthesiometer) Hypoesthesia index	Subjective perineal numbness (participants no): 11 vs 2 Objective perineal numbness: Dorsal penis: 11 vs 3 Anterior scrotal: 8 vs 4 (non-significant) Posterior scrotal: 9 vs 8 (non-significant) Hypoesthesia index: 3.43 vs 1.86

### Standing Versus Sitting

**Fig. 4:**
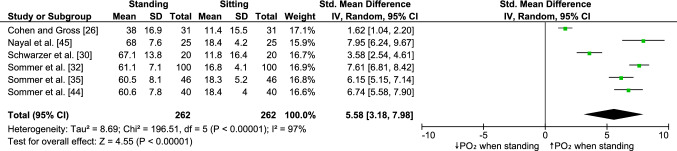
Forest plot showing the effect of standing vs sitting on the saddle on penile oxygen pressure; *PO*_*2*_ penile oxygen pressure

Six studies examined the effect of sitting on the saddle on transcutaneous penile oxygen pressure (Fig. 4). One study examined the effect of this intervention on penile blood flow measured using a laser Doppler flowmeter, and one measured penile blood pressure. On average, sitting on the saddle reduced transcutaneous penile oxygen pressure by 72.58%. The meta-analysis showed a significant (*P* < 0.00001, Z = 4.55) effect size of 5.58 (95% CI 3.18, 7.98). Consistently, Jeong et al. [22] have shown a significant reduction (1.7–1.0 ml/min/100 g tissue) of penile blood flow. Kerstein et al. [23] examined the effect of sitting on the saddle on penile blood pressure. They have shown that penile blood pressure decreased from 126 to 76 mmHg after 5 min of sitting on the saddle. After a 10-min recovery period, penile blood pressure returned to normal values.

### Different Saddle Designs

Various saddle designs were examined in 12 of the included studies. Saddles can be divided into three broad categories: (1) standard, narrow saddles without any cutouts or depressions, (2) saddles with central cutout or depression, (3) saddles without the nose.

Two studies examined the effect of using seats with central cutout on the total seat pressure [24, 25]. Both reported a non-significant increase of overall seat pressure compared with a standard saddle (30.3 kPa vs 27.6 kPa and 2.09 Pa/kg vs 2.04 Pa/kg for central cutout vs standard saddle). Bressel et al. [25] additionally measured anterior and posterior seat pressures. Using the holed saddle was associated with a significant reduction in anterior seat pressure (31 kPa vs 26.8 kPa) and an increase in posterior seat pressure (23.4 kPa vs 27.1 kPa). No significant difference in penile oxygen pressure between using standard and central cutout saddles was found [26].

Two studies compared the effects of standard saddles and saddles with nose cutout on the perineal pressure [27, 28]. The average reduction of perineal pressure using a nose cutout saddle (compared with standard saddle) was 63.24%. Use of the no-nose saddle resulted in a significant reduction in anterior (31 kPa vs 8.99 kPa), and a significant increase in posterior (23.4 kPa vs 27.3 kPa) seat pressures. No significant differences in total seat pressure were observed [25]. This is consistent with the results of Chen et al. [29]. They have examined the effect of different protruding node lengths of the seat on subjective discomfort levels in the perineum and ischial tuberosity. The result was a positive, significant correlation between protruding node length and discomfort in the perineum (*r* = 0.996). The correlation between protruding node length and discomfort in the ischial tuberosities was negative (*r* = − 0.914). Schwarzer et al. [30] showed that using a no-nose saddle resulted in a substantially smaller decrease of penile oxygen pressure (20.3%) when compared with a standard, narrow saddle (72.4%). Schrader et al. [28] examined the effect of the no-nose saddle on perineal pressure, urogenital numbness, and Index of Erectile Function Questionnaire (IIEF) score. They showed that a no-nose saddle reduced occurrence of urogenital numbness over 6 months from 73 to 18%. They also showed a small, but significant increase in the IIEF score. Parthiban et al. [31] examined the effect of using the no-nose saddle on occlusion time proportion (OTP). First, they measured the minimal force required to occlude perineal arteries in various anatomical points. As a next step, force sensors were placed on the perineum. The OTP was defined as the proportion of the total ride time when any sensor reached force required for occlusion of the corresponding perineal artery. A no-nose saddle reduced OTP by 0.23 compared with a standard saddle. Sommer et al. [32] and Schwarzer et al. [30] examined the effect of a saddle with no-nose on penile oxygen pressure. They showed that reduction of penile oxygen pressure caused by sitting on the no-nose saddle was considerably smaller compared with reduction caused by sitting on the standard, narrow saddle (20.3–22.3% for the no-nose saddle versus 70.4–82.4% for the standard saddle). Munarriz et al. [20] reported an examination of the right and left CAPVS in patients with erectile dysfunction, which was suspected to be caused by bicycle riding. Using the no-nose saddle resulted in significantly higher values of right/left CAPSV (0.36/0.77 cm/s vs 21.57/21.13 cm/s). Two studies examined the stability of various seats. Chen et al. [23] have shown, that subjective rating of stability was significantly lower for seats with short protruding node lengths than for classic seats with a long nose (4.36 vs 2.61 on VAS; lesser values signify higher perceived stability). A similar reduction in the perceived stability for no-nose seats has been shown by Bressel et al. [21].

Jeong et al. [22] explicitly compared wide and narrow saddles. They showed an 84% decrease of penile blood flow for the narrow saddle and a 19% decrease for the wide saddle. Schwarzer et al. and Sommer et al. also performed a comparison of wide and narrow saddles [30, 32]. They showed—respectively—63.6% and 63.3% reduction of penile oxygen pressure. Taylor et al. [33] examined experimental saddle design with a cutout in the nose. They proved that experimental design was associated with a significant reduction of perineal numbness (11 participants experiencing perineal numbness when using standard saddle versus 2 participants when using experimental design, *P* < 0.01). Breda et al. [34] compared partial penile oxygen pressure when using an ordinary, narrow saddle against an SMP saddle, designed specifically with perineal protection in mind. They have shown that using an SMP saddle resulted in significantly higher values of penile oxygen pressure (49.3 vs 25.3 mmHg after 3 min of static sitting, 52.1 vs 28.5 mmHg after 15 min of pedaling).

### Different Positions

Sommer et al. compared cycling in two different positions—upright and reclining. The reclining position was associated with a non-significant reduction in penile oxygen pressure from 61.1 to 59.4 mmHg, while the upright position was associated with a significant reduction from 60.5 to 18.3 mmHg [35]. Carpes et al. [24] tested the effect of upright and forward (trunk angle 90° and 60°) positions on total seat pressure using two different saddle designs (with and without a hole). They have shown that 60° trunk position results in significantly smaller seat pressure (55.75 vs 66.3 for 90°), only when using a holed saddle. When using a plain saddle, the difference in total seat pressure between two different positions was not significant. Bressel et al. [36] tested the effect of the top handlebar and drop handlebar positions on anterior, posterior, and total seat pressures. There were no significant differences in pressures in any of these regions. Potter et al. [37] examined the effect of the top and drop handlebar positions on anterior and posterior seat pressures. Using tops handlebar resulted in significantly greater posterior seat pressure (0.576 vs 0.392 kPa/kg; pressure normalized to bodyweight). The difference in anterior seat pressure was not significant. Chen et al. [29] reported that using tops or drops handlebars did not significantly influence reported discomfort in the perineum or ischial tuberosity region.

### Other Strategies

Sanford et al. [38] evaluated the effect of a seat shock absorber on perineal pressure in various conditions—pedaling or stationary, with and without artificial oscillations. A shock absorber significantly reduced pressure in the anterior and posterior perineum in stationary conditions with oscillations but not while pedaling with oscillations. Marcolin et al. tested [39] the impact of shorts with three different pads—basic model, designed for short distances, intermediate model, and an endurance model developed for longer distances. The differences in perineal pressure were not significant (12.6 kPa vs 12 kPa vs 12.3 kPa). Sommer et al. [32] tested if a recumbent bike reduced the impact of cycling on transcutaneous penile oxygen pressure. Cycling on a recumbent bike resulted in a substantially smaller reduction of penile oxygen pressure (2.13%) when compared with an ordinary bike (72.5%).

## Discussion

The main goal of our systematic review and meta-analysis was to examine the effectiveness of various strategies aimed at reducing the impact of cycling on the perineum. Since a reduction in the pressure in one region of the seat might be associated with increased pressure in another, we have additionally examined pressures and comfort in other regions in contact with the seat. The strategies we have obtained included different saddle designs, changes of position, shorts with various types of pads, a saddle shock absorber, and using a recumbent bike.

### Different Types of Saddle

At least a 60% reduction of anterior seat pressure is necessary to significantly decrease internal perineal compression [40]. Using the no-nose saddle resulted in a 71% reduction, which was associated with an increase of penile oxygen pressure, reduction of the discomfort in the perineum, and a smaller incidence of perineal numbness. However, this kind of seat was associated with increased posterior seat pressure and greater discomfort in the ischial tuberosities. Additionally, no-nose seats were rated as less stable than conventional seats with a long, protruding nose.

Using a central cutout saddle resulted in a non-significant increase in total, a significant decrease in anterior, and a significant increase in posterior seat pressures compared with a standard saddle [25, 41]. An observational study by Dettori et al. [7] has shown that using cutout saddle is associated with a slightly greater risk of erectile dysfunction among cyclists with perineal numbness. They attribute this increased risk to vulnerable anatomical variants among cyclists with perineal numbness and to the edges of the cutout which increased the pressure applied to the perineum. More recent evidence hints that anatomical variants may indeed play a role—Nanka et al. [11, 42] report that sulcus nervi dorsalis penis varies in depth from 0 to 2 mm. They hypothesize that a deeper sulcus plays a protective role in cycling-induced sexual dysfunction.

### Position

Our results suggest that the cyclist’s position may nullify the potential benefits of using saddles with central depression. Riding in the 60° trunk position when using a holed saddle resulted in significantly smaller total seat pressure. This result may not translate to perineal protection. Firstly, the reduction of total seat pressure may not necessarily result in a reduction of perineal or anterior seat pressure. Secondly, three-dimensional models estimated available space between the seat and pubic symphysis as follows: 52 mm^2^ for a rider in a fully forward position and 73 mm^2^ for cyclists sitting upright for grooved seats [12]. With this in mind, we suspect that even though total seat pressure was higher, the pressure on the anterior perineum may be lower when using an upright position. Current evidence on the topic is inconclusive.

When using a standard saddle, there was no significant difference between total seat pressure when riding with a trunk angle of 60° and 90°. Lack of difference between riding in either the 60° or 90° position on anterior seat pressure is indirectly supported by results regarding hands position on the handlebar. Typically, a cyclist using the top handlebar position sits with a trunk angle closer to 90°, and when using drop handlebar position closer to 60°. Two of the included studies reported no significant difference between the top and drop handlebar position in anterior seat pressure. There was a significant increase in posterior seat pressure when using the top handlebar position, but this did not influence reported discomfort in the perineum or ischial tuberosity region.

### Other Strategies

There is limited evidence regarding other strategies. Only one study examined the effect of a seat shock absorber on perineal pressure. The strategy was effective in reducing perineal pressure. Current evidence does not support (or refute) the use of different models of pads for perineal protection.

Our results hint that using the recumbent bike is an effective strategy in reducing the impact of cycling on the perineum. Using a recumbent bike mitigated decrease of penile oxygen pressure associated with using a standard bike and seat. A recumbent bike is typically used in a reclining position, which reduces the impact of cycling on penile oxygen pressure.

### Practical Implications and Limitations

Our paper has several limitations. First, the majority of included studies were performed in a laboratory setting. This approach omits several important conditions associated with cycling in the field setting. The notable examples include different terrain, oscillations, and varying workloads, which could affect the perineal pressure and comfort of the bicycle user [38, 41]. Additionally, most of the included studies consisted of just a few, relatively short sessions. Another shortcoming is the frequent use of various pathophysiological measures such as penile oxygen pressure or perineal pressure without relating them to clinical outcomes such as perineal numbness or IIEF score. Poor riding technique and incorrect bicycle fit are suspected to be common causes of genital numbness. A case report of two cyclists showed significant improvement of genital numbness after the correction of these factors [43]. For this reason, we consider the lack of interventional studies examining the effect of correcting the bicycle fit and posture of the bicycle user on the perineum as a limitation of our manuscript.

While using the recumbent bike resulted in higher penile oxygen pressure when compared with the standard bike, some issues limit their popularity. Firstly, due to their aerodynamic advantage, the use of recumbent bikes has been banned from a wide range of cycling races. Secondly, the rider in the recumbent bike is significantly lower compared to a standard bike. This leads to a reduction in the visibility of traffic and reduced visibility of the cyclist. Current evidence does not refute (or validate) these safety concerns.

Schrader et al. [28] identified three main concerns related to using no-nose saddles expressed by cyclists: a shift of weight distribution from the saddle to the handlebar, worse bicycle handling, and fear of sliding forward from a saddle causing blunt trauma. We have identified another limitation of using a no-nose saddle—an increase in posterior seat pressure. Evidence regarding handlebar pressure is conflicting and summarized by Schrader [28]. The data concerning safety are very scarce. While out of 85 police officers using the no-nose saddle for six months none suffered blunt trauma caused by sliding off the saddle, it is important to point out that this study population is insufficient to accurately determine the true incidence rate. Schrader [28] reports that out of 90 officers, only three returned to using the standard saddle after six months. While this result is promising, the design of the study (i.e. before and after trial) is likely to introduce significant bias in this area. Two of the included studies examined the stability of no-nose saddles. Both reported that using them was associated with lower perceived stability. However, these results come from short-term studies. It can not be ruled out that with longer use and experience stability scores would improve.

Standing on the pedals every ten minutes is frequently mentioned as a safe and easily applicable strategy for reducing the impact of cycling on the perineum [44]. Currently, there is no direct evidence supporting this strategy. However, it is indirectly supported by several results from the studies included in our manuscript. The strongest and most consistent result we have obtained is that riding in the standing position negates the negative effect of cycling on penile oxygen pressure. Additionally, an observational study by Awad et al. reported that standing more than 20% of the duration of the ride reduced the odds of genital numbness [4]. This strategy introduces several variables that are yet to be explored in depth. For example, it is not clear how long a cyclist should remain in the standing position for penile oxygen pressure to return to the norm (however, as Sommer et al. report it is ten minutes or less [35]). It is also not clear how often one should get up from his seat—10 min is an arbitrary number for which we did not find justification in current literature. Because standing on the pedals from time to time is a very safe intervention, we believe that it could be a viable, easily applicable strategy in reducing the impact of cycling on the perineum, but more research is needed to validate its effectiveness and to develop optimal guidelines.

## Conclusions

Current evidence supports the use of no-nose saddles as a means to reduce the negative impact of cycling on penile oxygen pressure. Standing on the pedals every ten minutes might be an effective and potentially widely applicable strategy. The use of a recumbent bike appears to protect the perineum, but several concerns prevent their widespread use.

## Electronic supplementary material

Below is the link to the electronic supplementary material.

Supplementary file1 (DOCX 415 kb)
