# Supplementary material for: Strategies for Reducing the Impact of Cycling on the Perineum in Healthy Males: Systematic Review and Meta-analysis
Source: Sports Med. 2020 Oct 19;51(2):275–87. doi: 10.1007/s40279-020-01363-z (PMC7846539; doi:10.1007/s40279-020-01363-z)
Supplement: Supplementary file 1 — Supplementary file1 (DOCX 415 kb) [file 40279_2020_1363_MOESM1_ESM.docx]

Article title: Strategies for reducing the impact of cycling on the perineum in healthy males: systematic review and meta-analysis.

Journal name: Sports Medicine

Author names: Kamil Litwinowicz, Marcin Choroszy, Anna Wróbel

Corresponding author:

Kamil Litwinowicz*, MD

Department of Medical Biochemistry,

Wroclaw Medical University,

ul. Chalubińskiego 10,

50-368 Wroclaw

E-mail: kamil.litwinowicz@student.umed.wroc.pl


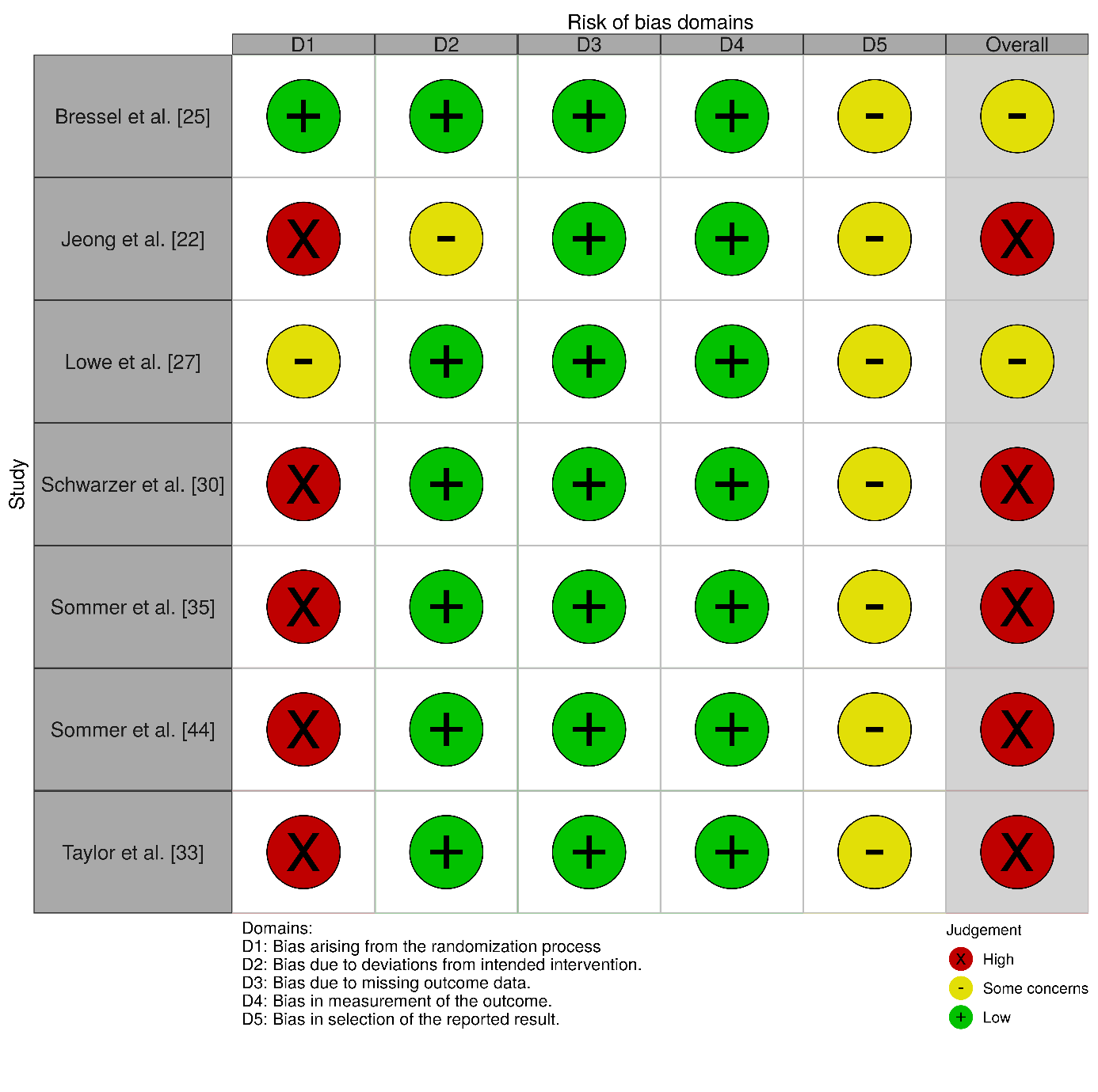


**Electronic Supplementary Material Fig. S1.** Risk of bias assessment of cross-over and randomized trials


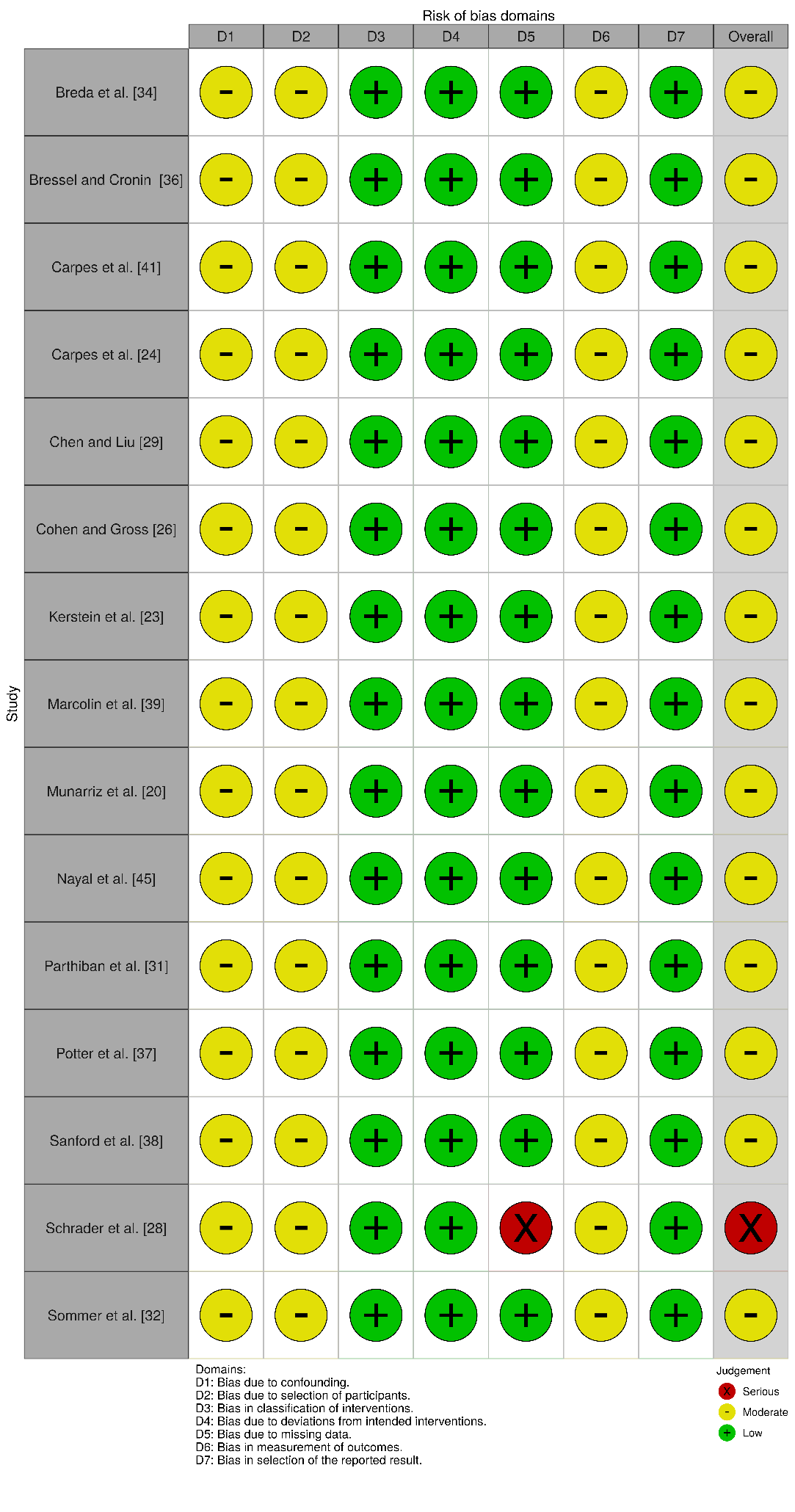


**Electronic Supplementary Material Fig. S2.** Risk of bias assessment of before and after studies

**Declarations:**

**Availability of data and material**: Not applicable

**Code availability**: Not applicable

**Authors' contributions**: All authors contributed to the study conception and design. Literature search and data analysis were performed by Kamil Litwinowicz, Marcin Choroszy and Anna Wróbel. The first draft of the manuscript was written by Kamil Litwinowicz and all authors commented on previous versions of the manuscript. All authors read and approved the final manuscript.

**Compliance with ethical standards**

**Ethics approval**: Not applicable

**Consent to participate**: Not applicable

**Consent for publication:** Not applicable

**Funding:** The authors received no specific funding for this work.

**Conflict of interest:** Kamil Litwinowicz, Marcin Choroszy and Anna Wróbel declare that they have no conflict of interest.
